# How the Brain Signals a Sound Source

**DOI:** 10.1371/journal.pbio.0030103

**Published:** 2005-02-22

Navigating one's environment requires sensory filters to distinguish friend from foe, zero in on prey, and sense impending danger. For a barn owl, this boils down mostly to homing in on a field mouse scurrying in the night. For a human—no longer faced with the reputedly fearsome saber-toothed Megantereon—it might mean deciding whether to fear rapidly approaching footsteps from behind on a dark, desolate street.

How does the brain encode auditory space? The long-standing model, based on the work of Lloyd Jeffress, proposes that the brain creates a topographic map of sounds in space and that individual neurons are tuned to particular interaural time differences (difference in the time it takes for a sound to reach both ears). Another key aspect of this model is that the location of a sound source is encoded by the identity of responding neurons.[Fig pbio-0030103-g001]

**Figure pbio-0030103-g001:**
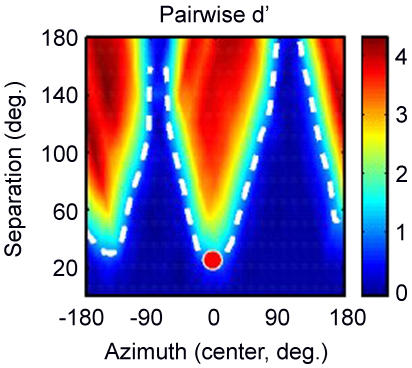
Discriminating sound locations from neural data

Evidence for local coding of auditory space has been shown in the brains of owls and in a subcortical region of small mammals, but no such map has been found in the higher centers of the mammalian auditory cortex. What's more, electrophysiological recordings in mammals indicate that most neurons show the highest response to sounds emanating from the far left or right and that few neurons show that kind of response to sounds approaching head-on—even though subjects are best at localizing sounds originating in front of them.

Faced with such contrary evidence, other investigators have suggested that sound localization may rely on a different kind of code—one based on the activity distributed over large populations of neurons. In a new study, Christopher Stecker, Ian Harrington, and John Middlebrooks find evidence to support such a population code. In their alternative model, groups of neurons that are broadly responsive to sounds from the left or right can still provide accurate information about sounds coming from a central location. Although such broadly tuned neurons, by definition, cannot individually encode locations with high precision, it is clear from the authors' model that the most accurate aural discrimination occurs where neuron activity changes abruptly, that is, at the midpoint between both ears—a transition zone between neurons tuned to sounds coming from the left and those tuned to sounds coming from the right. These patterns of neuronal activity were found in the three areas of the cat auditory cortex that the authors studied.

These findings suggest that the auditory cortex has two spatial channels (the neuron subpopulations) tuned to different sound emanations and that their differential responses effect localization. Neurons within each subpopulation are found on each side of the brain. That sound localization emerges from this opponent-channel mechanism, Stecker et al. argue, allows the brain to identify where a sound is coming from even if the sound's level increases, because it is not the absolute response of a neuron (which also changes with loudness) that matters, but the difference of activity across neurons.

How this opponent-channel code allows an animal to orient itself to sound sources is unclear. However auditory cues translate to physical response, the authors argue that the fundamental encoding of auditory space in the cortex does not follow the topographic map model. How neurons contribute to solving other sound-related tasks also remains to be seen.

